# Mitotically heritable effects of BMAA on striatal neural stem cell proliferation and differentiation

**DOI:** 10.1038/s41419-019-1710-2

**Published:** 2019-06-17

**Authors:** Paula Pierozan, Oskar Karlsson

**Affiliations:** 1 Science for Life Laboratory, Department of Environmental Sciences and Analytical Chemistry, Stockholm University, 114 18 Stockholm, Sweden; 2 Department of Pharmaceutical Biosciences, Uppsala University, Box 591, 751 24 Uppsala, Sweden

**Keywords:** Mechanisms of disease, Embryonic stem cells, Neurological disorders

## Abstract

The widespread environmental contaminant β-methylamino-L-alanine (BMAA) is a developmental neurotoxicant that can induce long-term learning and memory deficits. Studies have shown high transplacental transfer of 3H-BMAA and a significant uptake in fetal brain. Therefore, more information on how BMAA may influence growth and differentiation of neural stem cells is required for assessment of the risk to the developing brain. The aim of this study was to investigate direct and mitotically inherited effects of BMAA exposure using primary striatal neurons and embryonic neural stem cells. The neural stem cells were shown to be clearly more susceptible to BMAA exposure than primary neurons. Exposure to 250 µM BMAA reduced neural stem cell proliferation through apoptosis and G2/M arrest. At lower concentrations (50–100 µM), not affecting cell proliferation, BMAA reduced the differentiation of neural stem cells into astrocytes, oligodendrocytes, and neurons through glutamatergic mechanisms. Neurons that were derived from the BMAA-treated neuronal stem cells demonstrated morphological alterations including reduced neurite length, and decreased number of processes and branches per cell. Interestingly, the BMAA-induced changes were mitotically heritable to daughter cells. The results suggest that early-life exposure to BMAA impairs neuronal stem cell programming, which is vital for development of the nervous system and may result in long-term consequences predisposing for both neurodevelopmental disorders and neurodegenerative disease later in life. More attention should be given to the potential adverse effects of BMAA exposure on brain development.

## Introduction

Epidemiological and experimental studies have shown that unfavorable prenatal environmental factors, such as exposure to certain neurotoxic environmental contaminants, can have adverse consequences for neurodevelopment^1^. Evidence indicates developmental insults as potential causes of pathologies with long-term detrimental outcomes^2–4^. The sensitivity of the developing central nervous system has raised concerns about potential neurotoxic effects of compounds that fetuses and children are commonly exposed to^5^. Hundreds of chemicals have been detected in human umbilical cord blood, and since the blood brain barrier is not fully developed until 6 months postpartum, the chemicals may have easy access to the central nervous system^6,7^.

Harmful cyanobacterial blooms are increasing worldwide due to climate change and eutrophication^8,9^. One of the main issues with increasing amounts of cyanobacteria is their ability to generate secondary metabolites that are hazardous for humans. β-methylamino-L-alanine (BMAA) is a cyanobacterial toxin that has been detected in several water systems and found to accumulate in food chains^10–12^. BMAA is implicated as a possible risk factor for neurodegenerative diseases, particularly Guamanian amyotrophic lateral sclerosis/parkinsonism dementia complex (ALS/PDC)^13–15^. This neurotoxic amino acid has also been detected in the brains of ALS/PDC, Alzheimer’s disease, and ALS cases^13,14,16^. BMAA acts as an ionotropic and metabotropic glutamate receptor (mGluR) agonist that can induce neuronal degeneration mainly via excitotoxic mechanisms, at high concentrations in the millimolar range^17,18^.

The uptake of BMAA in discrete brain regions of rodent fetuses and neonates is higher and more selective than that in the brain of adults^19–21^. Autoradiographic imaging revealed high transplacental transfer of ^3^H-BMAA and a specific uptake in fetal mouse brain areas^22^. Moreover, BMAA treatment of neonatal rats induced transient behavioral changes, such as disturbed motor function and hyperactivity^22^ as well as long-term cognitive impairments^23,24^, changes in neuronal protein expression and intracellular fibril formation at adult age^25,26^. Developmental exposure to this neurotoxin can also alter several striatal neuropeptides that play a critical role in the development and survival of neurons^27^. However, more studies are needed to elucidate the effects of BMAA on developing brain cells.

Neural stem cells (NSCs) are self-renewing cells that can differentiate into neurons, astrocytes, and oligodendrocytes, and are vital for the formation of neural circuits during the pre- and post-natal periods^28^. Environmental effects on NSC homeostasis may consequently alter the development of neural structures and lead to impaired brain function. In vitro NSC cultures provide an excellent possibility for studying adverse effects of environmental contaminants on critical neurodevelopmental processes, including cell proliferation and differentiation. The proliferative ability of NSC also provides a tool for studying mitotically inherited effects^29^. Therefore, NSC represent a relevant in vitro model for mechanistic studies and toxicity assessment in the field of developmental neurotoxicology^30^.

In the current study, we have used primary neuronal cells and embryonic NSC from rat striatum to compare the susceptibility to the toxic effects of BMAA and determine the mechanistic role of glutamate receptors. We examine the effects of BMAA exposure on proliferation and spontaneous differentiation of NSC as well as potential effects on cell programming that could be inherited through mitosis.

## Material and methods

### Chemicals

β-N-methylamino-L-alanine hydrochloride (≥97% purity, CAS Number 16012-55-8; Sigma-Aldrich Co., St. Louis, MO, USA), paraformaldehyde, 4′,6-diamidino-2-phenylindole dihydrochloride (DAPI), Triton X-100, propidium iodide (PI), DNAse-free RNAse A, 3-(4,5-dimethyl-2-yl)2,5-diphenyl-2H-tetrazolium bromide (MTT), and basic fibroblast growth factor (bFGF) were obtained from Sigma-Aldrich (St Louis, MO, USA). Bovine serum, penicillin-streptomicin, Dulbecco’s phospate-buffered saline (PBS), Dulbecco’s modified eagle’s medium (DMEM), neurobasal medium, poli-ornithine, fibronectin, trypsin solution (0.05%), glutamine, and B27 were obtained from Gibco (Invitrogen, Paisley, UK). The secondary antibodies Alexa-Fluor 555 goat anti-mouse IgG (Cat #A32727), 488 goat anti-rabbit IgG (Cat #A32723), anti-goat alexa 350 (Cat #A11045), anti-chicken alexa 647 (Cat #A32728), the blocking agent (normal goat serum), and the Annexin–PI kit (Cat V13242) were obtained from Molecular Probes, Invitrogen (Paisley, UK). MK-801, 6-Cyano-7-nitroquinoxaline-2,3-dione (CNQX), and (RS)-α-methyl-4-carboxyphenylglycine (MCPG) were obtained from Tocris Bioscience (Bristol, UK). The antibodies β III-tubulin anti-rabbit (Cat ab18207), glial fibrilar acidic protein (GFAP) anti-mouse (Cat ab53554), and nestin anti-rabbit (Cat ab134017) were obtained from abcam (Cambridge, UK). The antibody oligo4 (Cat #1518925) was obtained from Chemicon (Temecula, CA, USA).

### Animals and housing

Pregnant outbred Wistar rats, obtained from Charles River (Sulzfeld, Germany), were housed alone in a Macrolon cage (59 × 38 × 20 cm) containing wood-chip bedding and nesting material. The dams were maintained on standard pellet food (R36 Labfor; Lantmännen, Kimstad, Sweden) and water ad libitum. The animals were housed in a temperature- and humidity-controlled environment on a 12-h light/dark cycle (lights on at 6:00 A.M.). All animal experiments were performed according to protocols approved by the Regional Animal Ethical Committee and in accordance with the Swedish Legislation on Animal Experimentation (Animal welfare act SFS1998:56) and the European Union Directive on the Protection of Animals Used for Scientific Purposes (2010/63/EU).

### Cell culture and BMAA exposure

Rats were euthanized by decapitation^31,32^ and primary neuronal cell cultures were prepared from striatum at embryonic day 18 as previously described^33^. In brief, single-cell suspensions were obtained by dissociating embryonic striatal cells in DMEM/F12 medium. Approximately 100,000/cm^2^ neuronal cells were plated on polylysine-treated 96-well plates. The neuronal cultures were kept in Neurobasal medium supplemented with 2 mM glutamine and B27 for up to 24 h. The culture medium was then replaced, and the cells were incubated for 7 days in a humid incubator (37 °C; 5% CO_2_). At 8 days in vitro (DIV), the culture medium was removed and cells were treated for 24 h with 50 µM to 3 mM BMAA dissolved in Neurobasal medium. Control cultures were incubated with Neurobasal medium only.

NSC cultures were prepared from rat striatum at embryonic day 15. The tissue was dissected and gently mechanically dispersed in Hank's Balanced Salt Solution, and the cells plated at density of 40,000/cm^2^ in T-75 flasks precoated with poly-L-ornithine and fibronectin. Cells were maintained in N2 medium enriched with 10 ng/mL bFGF, to keep cells in an undifferentiated state. Fresh bFGF was added daily and the complete medium was changed every other day. After 3 days in culture, cells were passaged after detachment by 0.05% trypsin-EDTA incubation. The cells were washed and mixed in N2 medium, plated at low density (500 cells/cm^2^) on plates coated with poly-L-ornithine and fibronectin, and grown in the presence of bFGF. One day after the passaging, cells were treated with 50 µM to 3 mM BMAA for 24 h. The cells were washed and N2 medium without any bFGF enrichment was added to promote spontaneous differentiation for 24 h (cell proliferation assays) or for 7 days in total (morphometric and differentiation assays). In the experiments designed to study signaling mechanisms, cells were preincubated with 100 μM MK-801, 25 μM CNQX, or 50 μM MCPG for 30 min and then coexposed with BMAA for 24 h. To investigate mitotically heritable effects in daughter cells (D1 and D2), parental (P1) NSCs were exposed for 24 h to 50 or 100 µM BMAA in N2 medium enriched with the differentiation inhibitor bFGF. The cells were then passaged (one passage for D1 and two passages for D2) by 0.05% trypsin-EDTA incubation, washing, centrifugation, mixed in N2 medium, and plated at low density (500 cells/cm^2^). Three days after passaging, bFGF was removed and the cells were allowed to differentiate for 24 h (cell proliferation assays) or 7 days. All experiments were performed using six replicates and repeated three times starting from preparation of cell cultures from new animals.

### Cell proliferative analysis

#### 3-(4,5-dimethylthiazol-2-yl)-2,5-diphenyltetrazolium bromide

Cell viability was measured by the MTT assay as previously described^33^. The cell viability was measured after 24 h BMAA exposure in P1 cells, and 24 h after passaging in the daughter cells. The formazan product generated during the incubation with 0.5 mg MTT was solubilized in dimethyl sulfoxide and measured at 490–630 nm using a Polarstar Optima microplate reader (Bmg Labtech, Offenburg, Germany).

#### Annexin V–PI labeling

The apoptotic/necrotic analysis was carried out by surface labeling with the Ca^2+^-dependent phosphatidylserine-binding protein annexin V and PI. Cells were recovered from the culture plates by 0.05% trypsin-EDTA treatment, centrifuged (1000 *g* for 5 min), and washed once with PBS. Cells were labeled by incubation with annexin V-FITC and PI in the provided binding buffer at room temperature for 15 min in the dark, according to the manufacturer’s instruction (Cat No. V13242, Invitrogen, Paisley, UK). Stained cells were analyzed (10,000 events) on a Cytoflex flow cytometer (Beckman Coulter Ltd., Brea, CA, USA).

#### Cell cycle analysis

Cells were processed for PI staining and flow cytometry as previously described^34^. Before analysis, the cells were detached from the culture plates with 0.05% trypsin/EDTA and centrifugated at 92 g for 5 min. Pelleted cells were then fixed by adding 2 mL 70% ice-cold ethanol dropwise while vortexing, and kept on ice for 1 h before storage at 4 °C. The samples were stored for at least 48 h before analysis to allow leakage of fragmented DNA from apoptotic cells and their identification as a fraction with DNA content less than G0/G1, referred to as the sub-G0/G1 fraction. On the day of analysis, fixed cells were kept on ice and washed twice in PBS, and each sample incubated in the dark with 1 mL PI (50 mg/mL) and RNAse A (50 ng/mL) in PBS for 3 h at 4 °C. Forward and light scatter data were collected in a linear mode. Fluorescence data for 10,000 cells per sample were collected in the FL3 channel on a linear scale. Side and forward light scatter parameters were used to identify the cell events and doublets cells were excluded using gating. Samples were analyzed using a Cytoflex flow cytometer (Beckman Coulter Ltd., Brea, CA, USA). Cells in different cell cycle phases were presented as a percentage of the total number of cells counted.

### Analysis of NSC differentiation

#### Immunocytochemistry

Immunocytochemistry was performed as previously described^33^. Briefly, cells were plated at density of 40,000/cm^2^ on microscope glass coverslips precoated with poly-L-ornithine and fibronectin and treated with 50 or 100 µM BMAA for 24 h. The cells were then fixed with 4% paraformaldehyde for 30 min and permeabilized with 0.1% Triton X-100 in PBS for 5 min at room temperature. After blocking, neurons were incubated overnight with anti-β III-tubulin (1:200) and anti-MAP2 (1:200), and NSC were incubated with β III-tubulin (1:200), anti-GFAP (1:500), anti-nestin (1:1000), and anti-oligo4 antibodies (1:1000) at room temperature, followed by three PBS washes and incubation with specific secondary antibodies conjugated with alexa 488 (sheep anti-rabbit, 1:1000) or with alexa 555 (sheep anti-mouse, 1:1000) for 1 h. The nucleus was stained with DAPI (0.25 mg/mL) before the cells were mounted and examined in an Olympus IX70 inverted microscope (Olympus, Tokyo, Japan). The images were collected by a CCD camera with 20× objective using constant intensity settings and exposure time for all samples. Semiquantitative analyses of differentiated cells were conducted in five randomly selected microscopic fields on each microscope slide. Images were analyzed with the ImageJ software (Sound Vision) after digital acquisition. In all immunostainings, negative controls reactions were performed by omitting the primary antibody. No reactivity was observed when the primary antibody was excluded.

#### Morphometric analysis

Cells were plated at density of 40,000/cm^2^ on 96-well plates precoated with poly-L-ornithine and fibronectin and treated with 50 or 100 µM BMAA for 24 h. Cells were then fixated and stained according to the immunocytochemistry section. After that, images were collected with a 10× objective in an ImageXpress Micro XLS Widefield High-Content Analysis System (Molecular Devices, Sunnyvale, CA, USA). Nine fields per well (~15,000 cells) were automatically analyzed with the SoftMax Pro Software after digital acquisition (Molecular Devices, Sunnyvale, CA, USA) using the MetaXpress Neurite outgrowth application module, based on β III-tubulin staining. The protocol was optimized for assessing cell morphology in our experimental conditions, including quantitative characterization of neural network complexity via several measurements such as total neurite outgrowth, number of processes and branches.

#### Flow cytometry

Cells were washed with PBS, recovered and dissociated with 0.05% trypsin-EDTA from culture plates and fixed in 4% paraformaldehyde for 10 min. After washing, the cells were permeabilized and blocked with 0.1% Triton X-100 and 1% BSA in PBS for 15 min. Then, 1,000,000 cells per sample were incubated with primary antibodies (anti-β III-tub, anti-GFAP, anti-nestin, and anti-Oligo4) at 4 °C overnight. After washing, cells were stained with secondary antibodies (anti-rabbit alexa 488, anti-goat alexa 350, anti-chicken alexa 647, and anti-mouse alexa 555) for 60 min at room temperature. Samples were analyzed using a Cytoflex flow cytometer (Beckman Coulter Ltd., Brea, CA, USA). The quadrants to determine the negative and positive area were placed on unstained and single stained samples. Forward and side light scatter gates were used to exclude cell aggregates and small debris. The number of cells in each quadrant was computed and the proportion of cells stained with β III-tub, GFAP, Oligo 4, and nestin were calculated.

### Statistical analysis

The results were presented as mean ± standard deviation for each experimental group consisting of at least three individual cell cultures, each with five to six replicates. The sample size was chosen based on a power of 0.8 and significance level of 0.05 using Minitab 15 software (MiniTab Inc, State College, PA, USA). No randomization of the samples was used and the experiments were performed blindly in all steps possible. Differences compared to the control group were analyzed by one-way analysis of variance (ANOVA) or by two-way ANOVA (signaling mechanisms experiments) followed by Tukey–Kramer multiple tests, or by Student’s *t*-test when comparing only two groups (cell cycle and flow cytometry) using Prism 7 (GraphPad Software, San Diego, CA, USA). The sample variation was similar between the statistically compared groups and all the data were normally distributed.

## Results

### Effect of BMAA exposure on neuronal viability and NSC proliferation

**Fig. 1:**
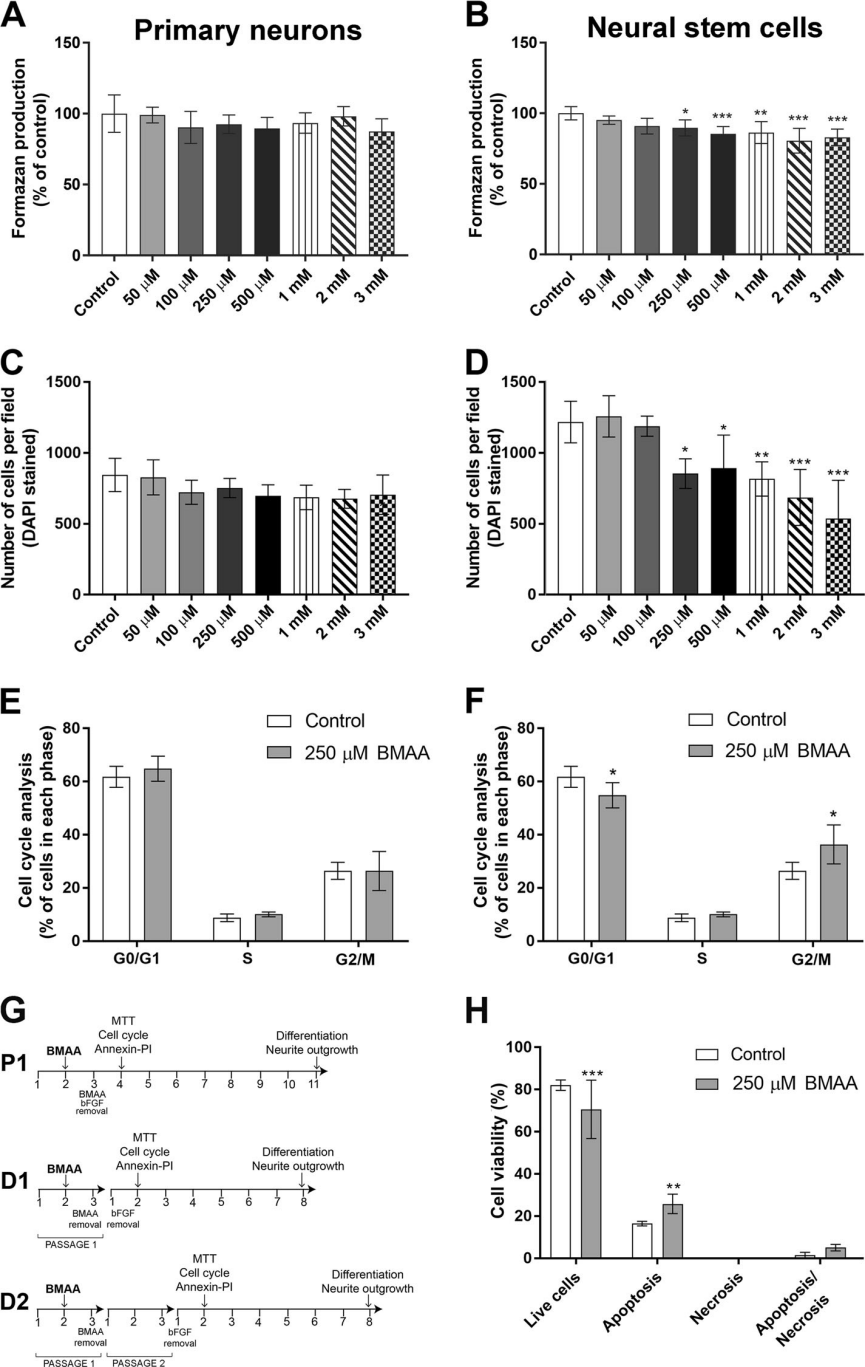
Effects of BMAA on cell viability and proliferation in striatal primary neurons and neural stem cells. Cell viability was determined by the MTT assay (**a**, **b**) and proliferation by DAPI staining (**c**, **d**) after treatment with 50 µM to 3 mM for 24 h. The cell cycle phase of cells treated with 250 µM BMAA was analyzed by flow cytometry (**e**, **f**). Apoptotic and necrotic cells were assessed with the annexin V and PI assay in cells treated with 250 µM BMAA (**h**). Apoptotic cell death was detected with 488-labeled annexin V; necrotic cells were detected with PI; cells that underwent apoptosis followed by necrosis were detected with annexin plus PI; and cells without labeling were live cells. The experimental design used for investigating BMAA effects on NSC (**g**). NSC were cultured for 3 days before passaging to obtain parent cells (P1). After 1 day in culture, P1 were exposed to BMAA for 24 h. To investigate mitotically inherited long-term effects of BMAA, P1 cells were passaged to daughter cells (D1 and D2). Values represent mean ± SD from three independent experiments, each with six replicates. Statistically significant differences from control are indicated as follow: ***p < 0.001; **p < 0.01 and *p < 0.05 (one-way ANOVA followed by Tukey–kramer test)

Primary neurons and NSC were incubated with 50 µM to 3 mM BMAA for 24 h to determine the effects on cell viability and proliferation. Controls were exposed to the cell medium only. The results show that BMAA exposure did not affect MTT production in primary neurons (Fig. 1a). However, in NSC the treatment induced a decreased MTT production at 250 µM to 3 mM BMAA (Fig. 1b). These results were confirmed by counting DAPI-stained cells (Fig. 1c, d). To determine effects on the cell cycle, cell cycle distribution was analyzed in primary neurons and NSC treated with 250 µM BMAA using flow cytometry. In accordance with the proliferation results the cell cycle in primary neuronal cells was shown not to be altered by BMAA exposure (Fig. 1e), while in the NSC cultures, the percentage of G0/1 phase cells was decreased and the percentage of G2/M phase cells was increased after BMAA treatment (Fig. 1f). Possible mechanisms of cell death caused by BMAA in NSC were explored using the Annexin–PI assay. We found a decreased number of viable cells and an increased number of cells in apoptosis in NSC treated with 250 µM BMAA compared to the control group (Fig. 1h).

### Mitotically inherited effects of BMAA on viability and proliferation

**Fig. 2:**
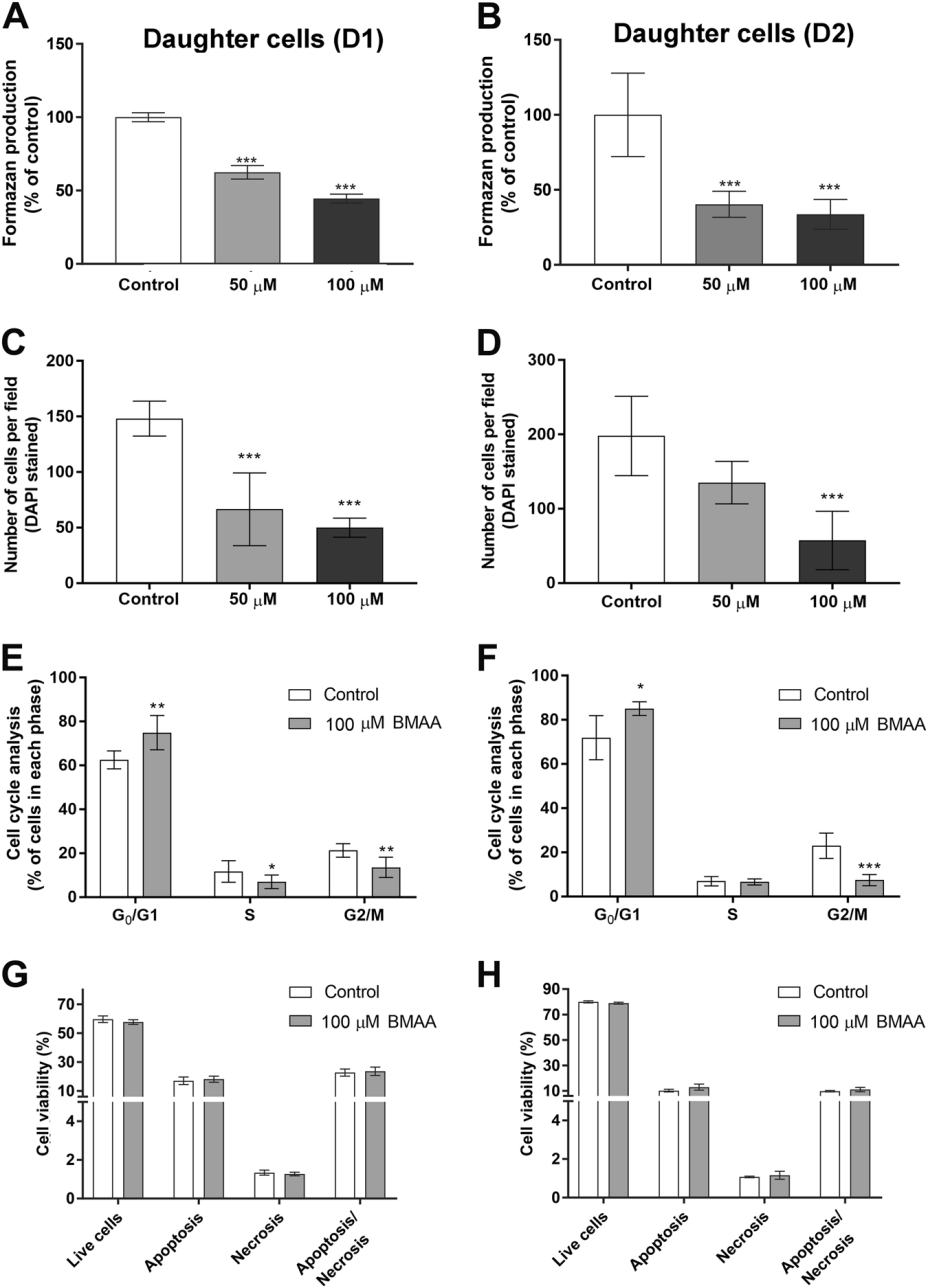
Effects of BMAA on cell viability and proliferation in daughter cells. Viability was determined by the MTT assay (**a**, **b**) and proliferation by DAPI staining (**c**, **d**) in daughter cells of NSC treated with 50 or 100 µM BMAA. The cell number was determined based on images of DAPI-stained cells on glass slides collected with a 20× objective according to the “Material and methods”. The cell cycle phase was analyzed by flow cytometry (**e**, **f**). Apoptotic and necrotic cells were assessed with the annexin V-488 and PI assay (**g**, **h**). Apoptotic cell death was detected with 488-labeled annexin V; necrotic cells were detected with PI; cells that underwent apoptosis followed by necrosis were detected with annexin plus PI; and cells without labeling were live cells. Values represent mean ± SD from three independent experiments, each with six replicates. Statistically significant differences from control are indicated as follow: ***p < 0.001; **p < 0.01 and *p < 0.05 (one-way ANOVA followed by Tukey–kramer test)

Heritable effects of BMAA exposure were investigated by analyzing the daughter cells of NSC exposed to noncytotoxic doses of BMAA (50 and 100 µM) (Fig. 1g). A decrease in MTT production was observed in the D1 and D2 passages cells compared to control (Fig. 2a, b). The reduction in cell proliferation was confirmed by counting DAPI-stained cells (Fig. 2c, d). Analysis of cell cycle distribution by flow cytometry revealed an accumulation of D1 and D2 cells in G0/1 phase, with concomitant decrease of the cells in G2/M phase (Fig. 2e, f), suggesting that BMAA alters cell programming in NSC that leads to mitotically heritable inhibition of the cellular proliferation through cell cycle inhibition. To confirm that the decrease in proliferation caused by BMAA is not due an induction of cell death, annexin–PI staining was conducted on the daughter cells. The results showed that neither apoptosis nor necrosis was increased in the D1 or D2 cells (Fig. 2g, h).

### BMAA exposure reduces NSC differentiation

**Fig. 3:**
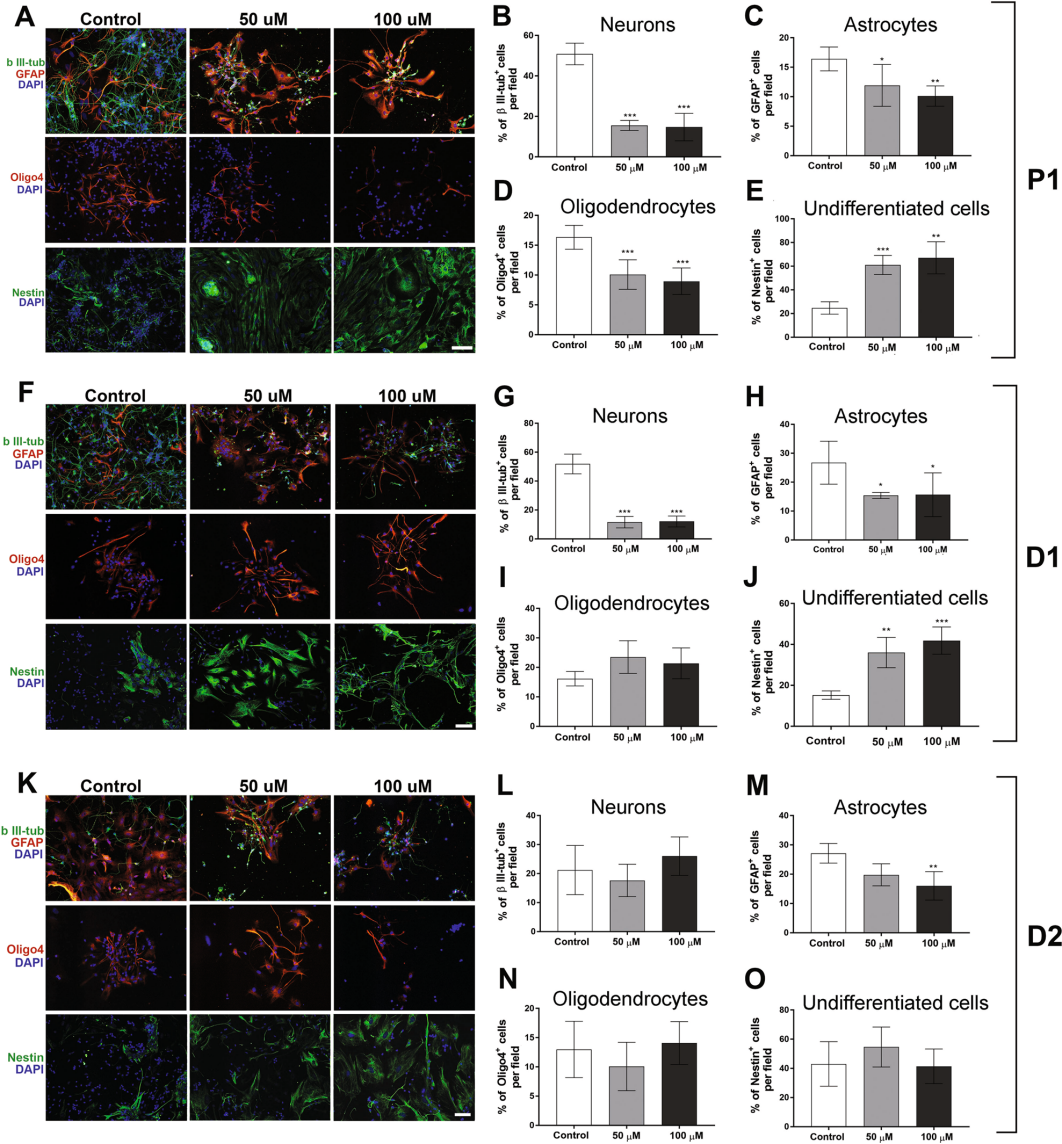
BMAA suppresses the differentiation of neural stem cells. NSC treated with 50 or 100 µM BMAA, and their daughter cells were fixed and stained with the neuronal marker β III-tubulin (upper panel, **a**, **f**, **k**, green) and astroglial marker GFAP (upper panel **a**, **f**, **k**, red); the oligodendrocyte marker Oligo4 (middle panel, red) and undifferentiated cells were marked with nestin (lower panel, green). The nuclei were stained with DAPI (blue). The slides were scored for the number of positive neurons (**b**, **g**, **l**), astrocytes (**c**, **h**, **m**), oligodendrocytes (**d**, **i**, **n**), and undifferentiated cells (**e**, **j**, **o**) considering fluorescence intensity and cellular morphology. Semiquantitative analysis for differentiated cells were conducted in five microscopic fields and expressed as mean ± SD from three independent experiments, each with six replicates. Statistically significant differences from control are indicated as follow: ***p < 0.001 and *p < 0.05 (one-way ANOVA followed by Tukey–kramer test). Scale bar = 30 µm

NSC differentiation were analyzed by immunohistochemistry after 24 h exposure to 50 or 100 µM BMAA, concentrations that did not induce cell death or proliferative alterations. The differentiation inhibitor bFGF was then removed to allow 7 days of spontaneous differentiation. After this, the cells were fixed and NSCs-derived neurons stained with a β III-tubulin antibody, astrocytes were stained with a GFAP antibody and oligodendrocytes with an oligo4 antibody. Undifferentiated cells were labeled with a nestin antibody. Representative immunofluorescence images are shown in Fig. 3a. A significant decrease in the percentage of NSC-derived neurons (Fig. 3b), astrocytes (Fig. 3c) and oligodendrocytes (Fig. 3d) were found in cells treated with 50 and 100 µM BMAA, while the percentage of undifferentiated cells was increased at both concentrations (Fig. 3e). The BMAA-induced effects on cell differentiation was confirmed by analyzing 10,000 cells with flow cytometry. NCS treated with 100 µM BMAA for 24 h demonstrated a reduction in the percentage of differentiated neurons, astrocytes, and oligodendrocytes, as well as an increase in the percentage of undifferentiated cells (Table 1).

**Table 1:** Effects of 100 µM BMAA on NSC differentiation in exposed (P1) and daugther cells (D1 and D2)

	Control	BMAA
P1		
Neurons	31.72 ± 3.61	16.66 ± 3.20***
Astrocytes	34.38 ± 3.25	15.49 ± 8.88**
Oligodendrocytes	15.75 ± 1.59	9.42 ± 3.3*
Undifferentiated cells	18.14 ± 5.78	58.44 ± 13.25***
D1		
Neurons	43.5 ± 1.37	28.69 ± 5.33**
Astrocytes	30.18 ± 3.45	16.62 ± 7.55*
Oligodendrocytes	16.21 ± 1.93	9.31 ± 2.5
Undifferentiated cells	10.57 ± 3.02	45.26 ± 11.44**
D2		
Neurons	25.58 ± 6.88	26.54 ± 9.24
Astrocytes	34.28 ± 3.7	18.32 ± 7.02**
Oligodendrocytes	9.52 ± 3.67	12.83 ± 0.82
Undifferentiated cells	30.61 ± 3.75	42.31 ± 8.97

Results are expressed as percentage of counted cells (10,000 events)

***p < 0.001; **p < 0.01 and *p < 0.05 (Student's *t*-test)

The mitotically inherited effects of BMAA on cell differentiation were investigated in daughter cells. Representative images of D1 and D2 cells are presented in Fig. 3f, k, respectively. The results revealed that the effects on NSC differentiation persisted in D1 (Fig. 3g–j and Table 1), while the only significant effect in D2 cells was a reduction in astrocytes (Fig. 3m and Table 1). No alterations in oligodendrocytes were observed in D1 and D2 (Fig. 3i, m).

### Effects of BMAA on morphological parameters of primary striatal neurons and NSC-derived neurons

**Fig. 4:**
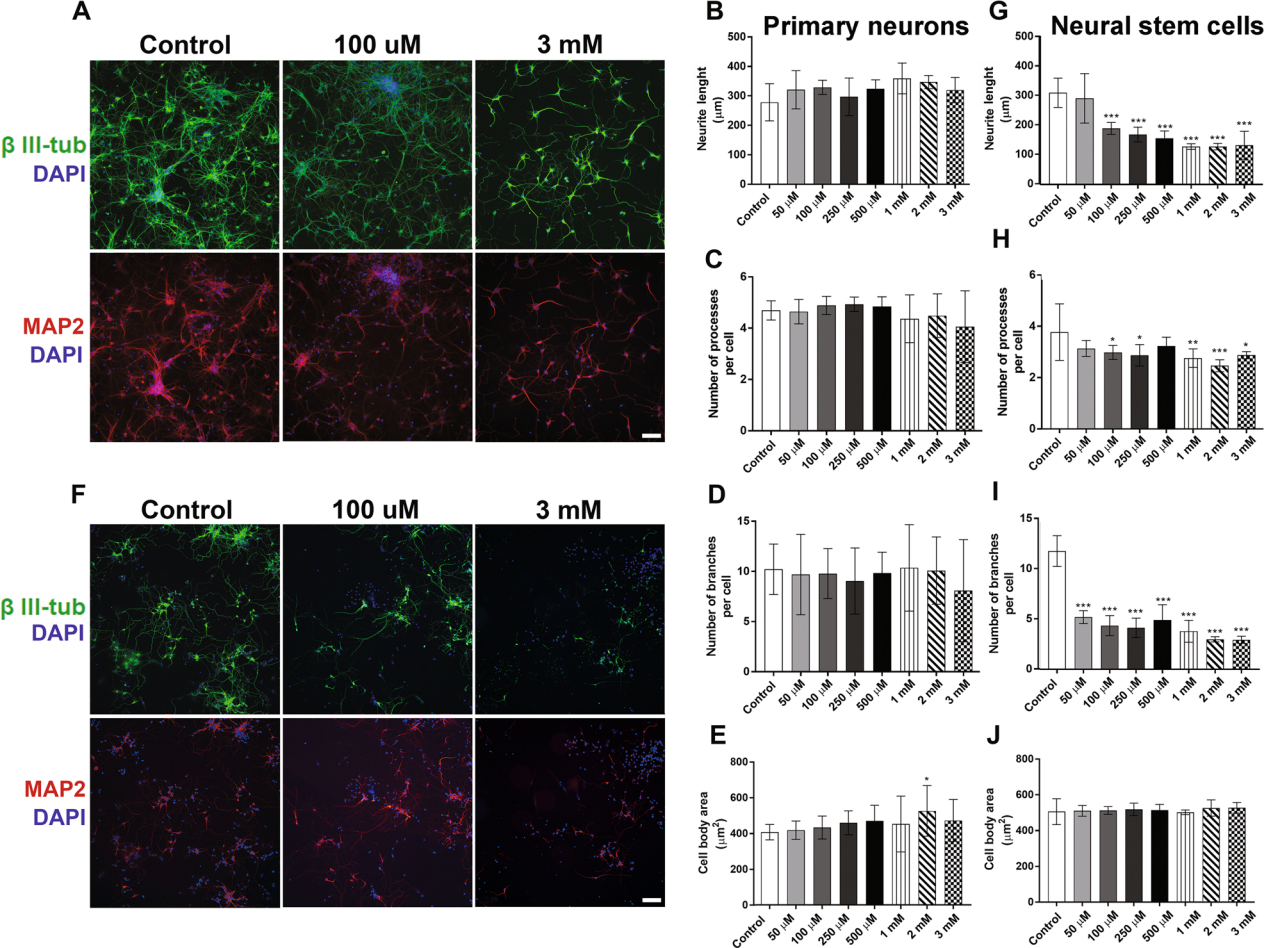
Morphometric alterations caused by BMAA on striatal primary neurons and neural stem cells. The effects on BMAA treatment with 50 µM to 3 mM BMAA was investigated directly after 24 h exposure of 8 DIV primary neurons, while the exposed NSCs were allowed to differentiate for 7 days after the exposure. Representative images of cells immunostained with anti-β III-tubulin (green), anti-MAP2 (red), and DAPI (blue) (**a**, **f**). Morphometric analysis was conducted using an ImageXpress Micro XLS Widefield HCA System (Molecular Devices, Sunnyvale CA, USA), where images were automatically captured and analyzed with the SoftMax Pro Software. Neurite length (**b**, **g**), processes per cell (**c**, **h**), branches per cell (**d**, **i**), and cell body area (**e**, **j**), were determined. Values represent mean ± SD from three independent experiments, each with five to six replicates. Statistically significant differences from control are indicated as follow: ***p < 0.001; **p < 0.01 and *p < 0.05 (one-way ANOVA followed by Tukey–kramer test). Scale bar = 50 µm

Since neurite outgrowth and the formation of synaptic contacts between neurons are essential for CNS development^35^, morphological parameters of primary striatal neurons treated with BMAA were evaluated. Immunocytochemical staining with 488-labeled anti-β III-tubulin and 555-labeled anti-MAP2 antibodies was conducted before images were automatically captured and analyzed using an ImageXpress Micro XLS Widefield HCA System (Molecular Devices, Sunnyvale CA, USA). The results showed that both controls and BMAA-treated neurons presented complex neurite meshworks containing long processes. BMAA exposure induced no morphological alterations in primary neurons except from a small increase in the cell body area at the 2 mM concentration only (Fig. 4a–e).

In contrast to primary neurons, BMAA induced large effects on the neurite development in NSC. Representative images are shown in Fig. 4f. The neurite length (Fig. 4g) and the number of processes per cell (Fig. 4h) were significantly reduced in cells treated with 100 µM to 3 mM. The number of branches per cell was significantly reduced even at the lowest concentration tested, 50 µM BMAA (Fig. 4i). No effects on cell body area was observed (Fig. 4j).

**Fig. 5:**
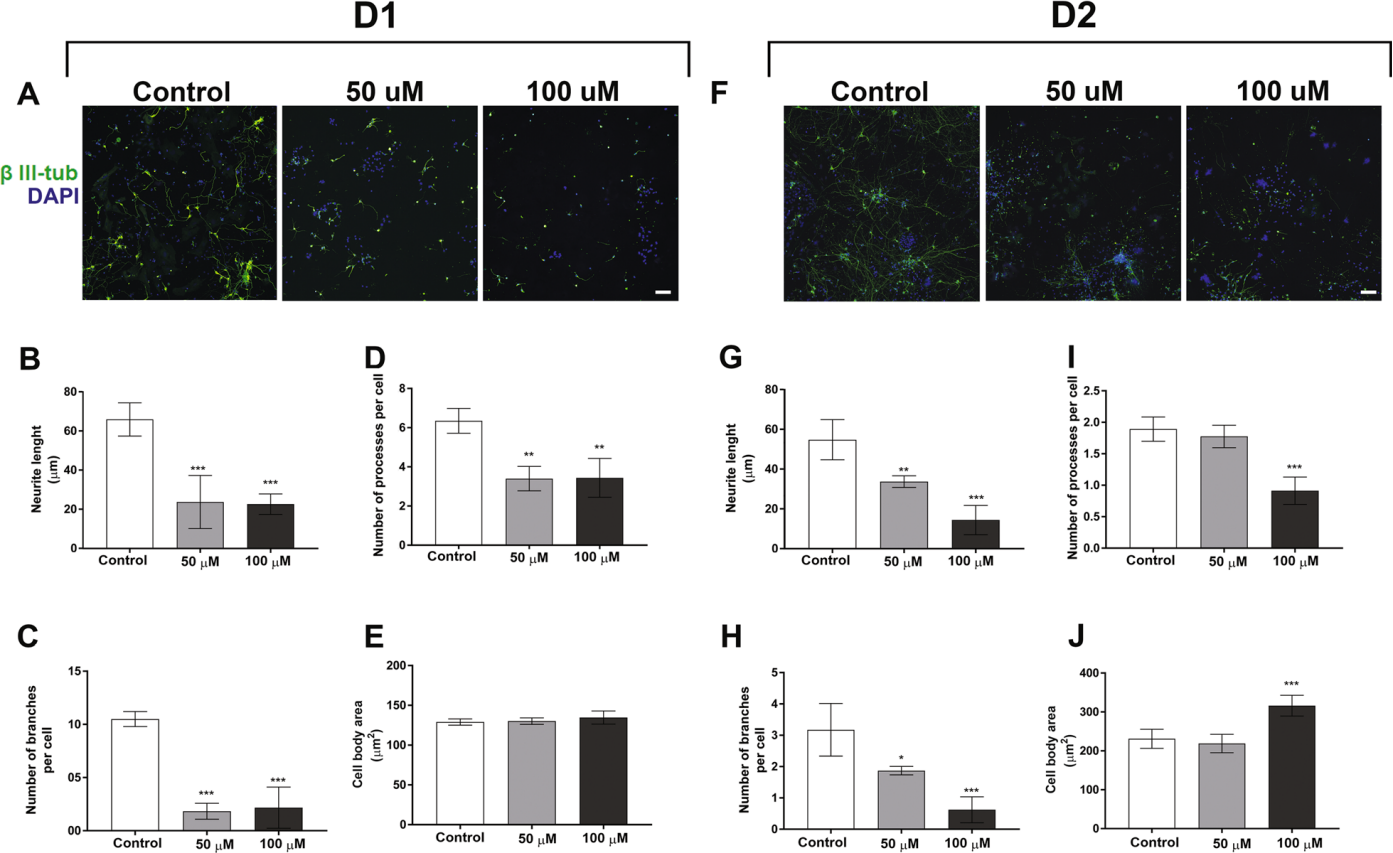
Morphometric alterations caused by BMAA in daughter cells. The effects on neuronal development were examined in daughter cells of NSC treated with 50 or 100 µM BMAA. Representative images of cells immunostained with anti-β III-tubulin (green) and DAPI (blue) (**a**, **f**). Morphometric analysis was conducted using an ImageXpress Micro XLS Widefield HCA System (Molecular Devices, Sunnyvale CA, USA), where images were automatically captured and analyzed with the SoftMax Pro Software. Neurite length (**b**, **g**), processes per cell (**c**, **i**), branches per cell (**d**, **h**), and cell body area (**e**, **j**) were determined. Values represent mean ± SD from three independent experiments, each with six replicates. Statistically significant differences from control are indicated as follow: ***p < 0.001; **p < 0.01 and *p < 0.05 (one-way ANOVA followed by Tukey–kramer test). Scale bar = 50 µm

The alterations in the neuronal differentiation caused by BMAA exposure of NSC persisted also in daughter cells after a multitude of cell divisions, and were actually more evident in D1 and D2 than in the parental cells exposed to BMAA. Figure 5a, f shows representative images of D1 and D2 passages of NSC treated with 50 or 100 µM BMAA. The morphometric analysis revealed a reduced neurite length (Fig. 5b, g), and a decreased number of processes (Fig. 5c, i) and branches per cell in the daughter cells (Fig. 5d, h). Cell body area was marginally altered, but only in D2 when parental cells were treated with 100 µM BMAA (Fig. 5e, j).

### The role of the glutamatergic system in BMAA-induced effects on NSC

**Fig. 6:**
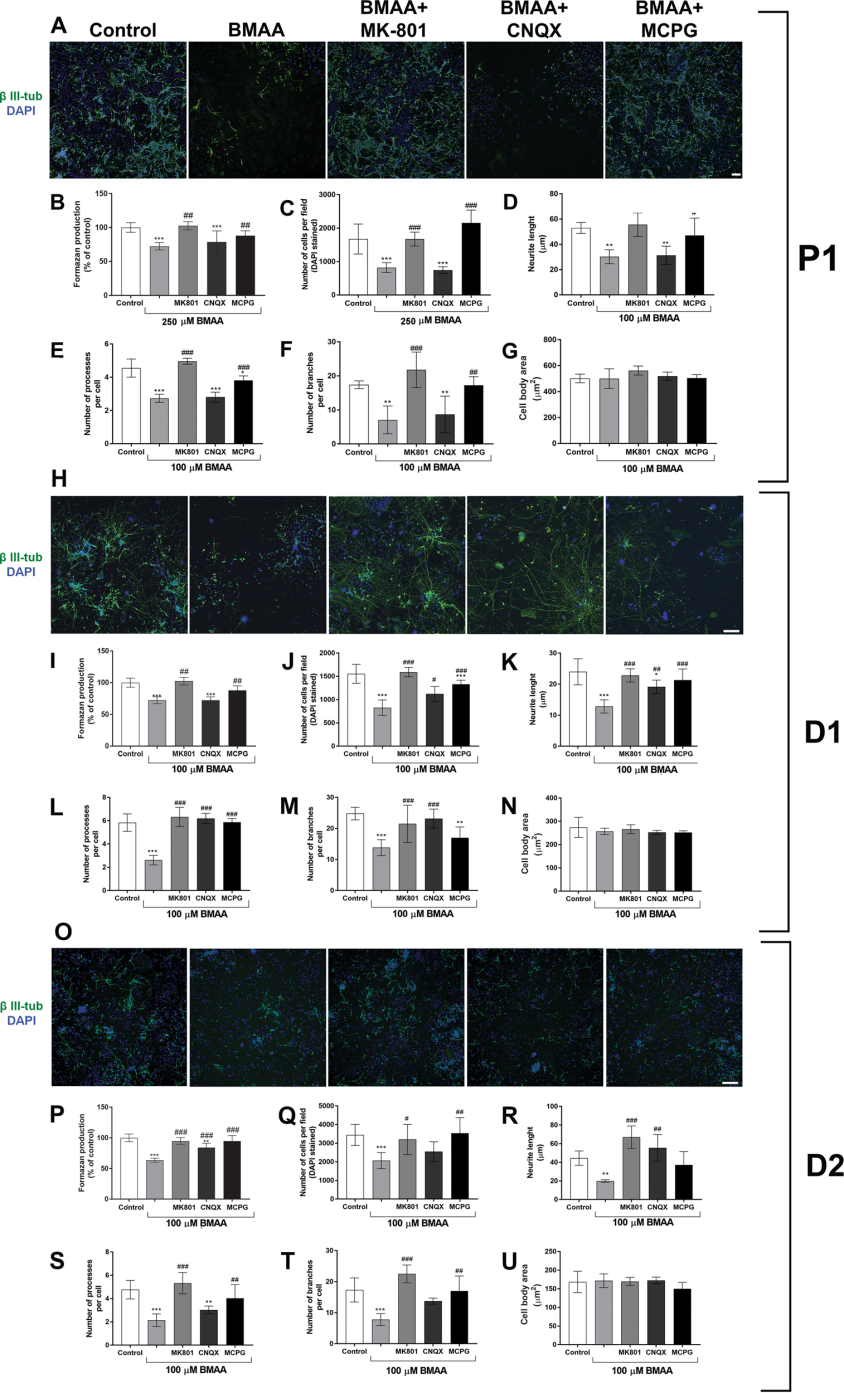
Glutamatergic mechanisms are involved in BMAA-induced proliferative and morphological alterations in neural stem cells. NSC were preincubated with glutamatergic antagonists (100 μM MK-801, 25 μM CNQX or 50 μM MCPG) for 30 min before cotreatment with BMAA for 24 h. Representative images of cells (**a**, **h**, **o**) stained with anti-β III-tubulin (green) and DAPI (blue) show that the glutamate antagonists MK-801 and MCPG, and partially CNQX, prevented the BMAA-induced neuronal alterations. Viability of the NSC treated with 250 µM BMAA, or daughter cells (D1 and D2) of NSC exposed to 100 µM BMAA, was determined by the MTT assay (**b**, **i**, **p**) and DAPI staining (**c**, **j**, **q**). The images were collected with a 10× objective in an ImageXpress Micro XLS Widefield High-Content analysis System and automatically analyzed with the SoftMax Pro Software according to the “Material and methods”. Morphometric analysis neurons show neurite length (**d**, **k**, **r**), processes per neurons (**e**, **l**, **s**), branches per cell (**f**, **m**, **t**), and cell body area (**g**, **n**, **u**) of neurons treated with the specific inhibitors and 100 µM BMAA. Data are reported as mean ± SD for three different experiments, each with six replicates, and analyzed by two-way ANOVA followed by Tukey–kramer test. Statistically significant differences are indicated as follow: ***p < 0.001 and **p < 0.01 (compared with control group); ^###^*p* < 0.001, ^##^*p* < 0.01, and ^#^*p* < 0.05 (compared with BMAA group). Scale bar = 50 µm

To investigate underlying pathways responsible for the effects triggered by BMAA in NSC, we examined the role of glutamate-mediated mechanisms. The ability of different glutamate antagonists to prevent the effects of the environmental toxin on cell viability (250 µM BMAA) and altered cell morphology (100 µM BMAA) was investigated. Figure 6a show representative images. The NMDA antagonist MK-801 and the metabotropic antagonist MCPG, but not the non-NMDA antagonist CNQX, completely protected against the decrease in cell viability (Fig. 6b) and proliferation (Fig. 6c) induced by BMAA in the NSC. In addition, MK-801 and MCPG prevented the effects caused by BMAA on the morphology of NSC-derived neurons, preserving the neurite outgrowth (Fig. 6d), number of processes (Fig. 6e), and number of branches per cell (Fig. 6f). The antagonists per se did not induce any alterations on these parameters (Supplemental Fig. 1).

When the parental cells were preincubated with the antagonists before exposure to 100 µM BMAA, MK-801, and MCPG completely prevented the reduction in cell number and morphologic alterations also in D1 and D2 cells (Fig. 6h–n, o–u). CNQX failed to prevent the cell viability in D1 and only partially prevented the reduction in cell number and number of branches per cells in D2 cells. The antagonists alone had no effects on the neuronal morphology (Supplemental Fig. 1).

## Discussion

Hazardous chemicals, which mainly may enter the body through contaminated food or water, pose a risk to the general population, and particularly to pregnant women and their unborn children. It is widely accepted that the developing brain is more sensitive to the harmful effects of environmental contaminants than the adult brain^36^. It is important to mechanistically elucidate BMAA's effects on the developing brain, as this toxin has been detected in water and food chains all over the world^37–39^, and is shown to adversely affect the immature brain^25,27,40–42^. The present study revealed different susceptibilities of primary striatal neurons and NSC to BMAA-induced toxicity. BMAA exposure at concentrations high as 3 mM had no effects on primary neurons, while concentrations in the micromolar range reduced cell proliferation, increased apoptosis, induced morphological changes, and altered cell differentiation in NSC, through glutamatergic mechanisms. Effects that were shown to be mitotically heritable.

The BMAA-induced decrease in NSC number was caused by cell cycle arrest and apoptosis. The cell cycle checkpoint at G2/M is critical in maintaining DNA integrity and regulating the passage of cells through the cell cycle, and the loss of these checkpoints is involved in the cell proliferation block and apoptosis^43^. Extracellular signals are important for the regulation of the cell cycle, and activation or inhibition of various checkpoint pathways by toxicants can alter the proliferation rate and cause cell death^44^. Our finding that BMAA caused cell cycle arrest and apoptosis through glutamatergic mechanisms are in line with several other studies demonstrating that overactivation of glutamatergic receptors and consequent increase of intracellular calcium can induce cell cycle changes and cell death^45–47^. However, previous studies of this toxin have shown that high BMAA concentrations—in the millimolar range—are necessary to induce neurodegeneration through glutamatergic mechanisms^48–50^. The demonstrated susceptibility of NSC to BMAA therefore confirms that early-life exposure to BMAA may be especially harmful. The results also provide evidence that compromised cell proliferation can be observed not only as a direct effect but also as mitotically inherited effect of BMAA exposure. Interestingly, in the daughter cells a cell cycle arrest in G0/G1 without any increase in cell death was observed. In accordance with this, previous studies have suggested that mechanisms of proliferative inhibition through G0/G1 arrest might be important in the maintenance of cell viability^51,52^.

The brain develops from a strip of cells along the dorsal ectoderm of the embryo into a complex organ consisting of billions of precisely located, interconnected, and specialized cells. Cell differentiation constitutes a crucial step in the development of CNS, and toxic interference of these processes could potentially lead to neurodevelopmental impairments. Our results showed that in addition to the adverse effects on cell proliferation and viability in NSC, BMAA specifically interfere with spontaneous neuronal, astrocytic, and oligodendrocytic differentiation even at the lowest concentration tested (50 µM). This effect is likely due to an inhibition of cell differentiation, as the results showed an increased ratio of undifferentiated cells compared with the control. Notably, BMAA also induced defects in the differentiation process in daughter cells. The finding suggests that BMAA induces long-term impairment of vital cellular pathways that can affect CNS development and may lead to neurodevelopmental disorders or predispose for brain disease later in life. Moreover, previous peptidomic analysis of the neonatal striatum after early-life BMAA exposure showed dose-dependent changes in neuropeptides such as enkephalins and neurosecretory protein VGF-derived peptides, which are critical for brain development^27^. The herein observed reduction of oligodendrocytes could indicate that BMAA induces effects on neurodevelopmental processes such as axonal growth and myelination. This is in line with a proteomic study demonstrating that neonatal exposure to BMAA decreased striatal myelin basic protein (MBP) at adult age^41^. MBP is essential for the formation and maintenance of myelin in the CNS, and demyelination and loss of oligodendrocytes are found in neurodegenerative diseases like Alzheimer disease^53^.

The present study revealed that BMAA exposure of NSC also decreased neurite outgrowth, the number of neurites and the number of branches per cells in NSC-derived neurons. These adverse effects on neuronal differentiation were mitotically inherited to daughter cells (D1 and D2), where the neurite meshwork impairments were even more evident than in the exposed cells. Neurite outgrowth is a key process during neuronal migration and differentiation, and the majority of neurons in mammalian brain are born and migrate to their destination site during embryonic development^54^. Moreover, to achieve mature function as transmitters of signals, the neurons must form connections by sending out dendrites and axons to form synaptic connections and the basic circuitries of the nervous system. Therefore, any inhibitory effect on neurite outgrowth can exert a neurotoxic effect on developing brain^55^. Importantly, the BMAA concentration shown to cause neurite alterations did not cause cell death or decreased cell proliferation, suggesting that the compound directly targets and reprograms pathways important for NSC neuronal differentiation and development of neurite morphology.

A variety of soluble and nonsoluble extracellular cues have been identified as important regulators of cell proliferation, differentiation, and axon development^56^. Glutamate regulates a variety of aspects of dendrite development, such as dendrite outgrowth, branching, and spine formation^57–59^. Sustained increase in intracellular Ca^2+^ concentrations caused by glutamate has also been linked with calpain-associated dendrite retraction^60^. The N-methyl-D-aspartate receptor (NMDAR) is highly expressed in the embryonic brain and involved in regulation of neurogenesis and neural development^61,62^, while the mGluRs play a major role in survival, proliferation, and differentiation of NSC^63,64^. This could explain why BMAA inhibits neurite outgrowth in developing neurons, but not in primary striatal neurons, through NMDAR and mGluR activation.

Early-life exposure to environmental chemicals particularly during intrauterine development has been suggested to program the risks for adverse health outcomes in adult life, including neurodegenerative disease^65,66^. There are a number of studies demonstrating that perinatal exposure of noxious agents alter the pattern of neural ontogenetic development and produce permanent neuroanatomical and neurochemical abnormalities^67^, which may be related to changes in genes that play a critical role in neural development, neural function and neurodegeneration^65,68^. Evidences indicate that epigenetic regulation including DNA methylation, histone modifications, and noncoding RNAs are involved in the developmental programming of late-onset pathologies^69^. Calcium entry through NMDAR results in activation of specific signaling pathways leading to changes in gene expression, affecting different epigenetic mechanisms like histone modifications and DNA methylation^70,71^. Enzymes controlling histone acetylation and methylation are involved in the whole process of neural development, regulating proliferation, promoting and inhibiting neurogenic, and gliogenic pathways as well as neurite outgrowth, and aberrant activity of these enzymes are associated with neural developmental disorders^72^. In addition, DNA damage (e.g., base modifications, basic sites, and strand brakes) caused by NMDA-generated ROS^73^ results in loss of NSC homeostasis and disturbs the balance between neurogenesis and gliogenesis^74,75^. These mechanisms may explain the BMAA-induced impairments in NSC differentiation and neurite outgrowth, and the mitotically heritable effects in daughter cells. However, further studies are needed to determine the detailed mechanisms underlying BMAA-induced NSC reprogramming.

In conclusion, the present study supports that BMAA, implicated as a possible risk factor for neurodegenerative disease, mainly acts as a developmental toxin. Primary striatal neurons were unsusceptible to BMAA concentrations as high as 3 mM, while NSC were significantly affected even at the lowest concentration tested (50 µM). BMAA exposure of NSC induced mitotically heritable alterations of several critical CNS developmental processes, including cell proliferation, differentiation, and neurite outgrowth. These effects could be related to the short- and long-term changes observed in rats developmentally exposed to BMAA, such as disturbed motor function, hyperactivity, striatal neuropeptide alterations, neurodegenerative pathology, and cognitive impairments in adulthood^22,27^. Considering the central role of NSC in the nervous system, the demonstrated alterations in NSC homeostasis may negatively impact the development of basic neuronal circuits and lead to deficits in brain function, including increased susceptibility for neurodegenerative disease later in life.

## Supplementary information

Supplemental Figure 1

## References

[1] Tamm C, Ceccatelli S (2017). Mechanistic insight into neurotoxicity induced by developmental insults. Biochem. Biophys. Res. Commun..

[2] Padmanabhan V, Cardoso RC, Puttabyatappa M (2016). Developmental programming, a pathway to disease. Endocrinology.

[3] Nathanielsz PW (2006). Animal models that elucidate basic principles of the developmental origins of adult diseases. ILAR J..

[4] Lyall K, Schmidt RJ, Hertz-Picciotto I (2014). Maternal lifestyle and environmental risk factors for autism spectrum disorders. Int. J. Epidemiol..

[5] Grandjean P, Landrigan PJ (2006). Developmental neurotoxicity of industrial chemicals. Lancet.

[6] Grandjean P, Landrigan PJ (2014). Neurobehavioural effects of developmental toxicity. Lancet Neurol..

[7] Risau W, Wolburg H (1990). Development of the blood-brain barrier. Trends Neurosci..

[8] Erdner DL (2008). Centers for oceans and human health: a unified approach to the challenge of harmful algal blooms. Environ. Health.

[9] Merel S (2013). State of knowledge and concerns on cyanobacterial blooms and cyanotoxins. Environ. Int..

[10] Banack SA, Johnson HE, Cheng R, Cox PA (2007). Production of the neurotoxin BMAA by a marine cyanobacterium. Mar. Drugs.

[11] Jiang L, Kiselova N, Rosen J, Ilag LL (2014). Quantification of neurotoxin BMAA (beta-N-methylamino-L-alanine) in seafood from Swedish markets. Sci. Rep..

[12] Li B (2019). Transfer of a cyanobacterial neurotoxin, beta-methylamino-l-alanine from soil to crop and its bioaccumulation in Chinese cabbage. Chemosphere.

[13] Murch SJ, Cox PA, Banack SA, Steele JC, Sacks OW (2004). Occurrence of beta-methylamino-l-alanine (BMAA) in ALS/PDC patients from Guam. Acta Neurol. Scand..

[14] Pablo J (2009). Cyanobacterial neurotoxin BMAA in ALS and Alzheimer’s disease. Acta Neurol Scand.

[15] Cox Paul Alan, Davis David A., Mash Deborah C., Metcalf James S., Banack Sandra Anne (2016). Dietary exposure to an environmental toxin triggers neurofibrillary tangles and amyloid deposits in the brain. Proceedings of the Royal Society B: Biological Sciences.

[16] Banack SA, Cox PA (2003). Biomagnification of cycad neurotoxins in flying foxes: implications for ALS-PDC in Guam. Neurology.

[17] Lobner D (2009). Mechanisms of beta-N-methylamino-L-alanine induced neurotoxicity. Amyotroph. Lateral Scler..

[18] Copani A (1991). Interaction between beta-N-methylamino-L-alanine and excitatory amino acid receptors in brain slices and neuronal cultures. Brain Res..

[19] Smith QR, Nagura H, Takada Y, Duncan MW (1992). Facilitated transport of the neurotoxin, beta-N-methylamino-L-alanine, across the blood-brain barrier. J. Neurochem..

[20] Karlsson O, Berg C, Brittebo EB, Lindquist NG (2009). Retention of the cyanobacterial neurotoxin beta-N-methylamino-l-alanine in melanin and neuromelanin-containing cells—a possible link between Parkinson-dementia complex and pigmentary retinopathy. Pigment Cell Melanoma Res..

[21] Karlsson O (2015). Environmental neurotoxin interaction with proteins: dose-dependent increase of free and protein-associated BMAA (beta-N-methylamino-L-alanine) in neonatal rat brain. Sci. Rep..

[22] Karlsson O, Lindquist NG, Brittebo EB, Roman E (2009). Selective brain uptake and behavioral effects of the cyanobacterial toxin BMAA (beta-N-methylamino-L-alanine) following neonatal administration to rodents. Toxicol. Sci..

[23] Karlsson O, Roman E, Berg AL, Brittebo EB (2011). Early hippocampal cell death, and late learning and memory deficits in rats exposed to the environmental toxin BMAA (beta-N-methylamino-L-alanine) during the neonatal period. Behav. Brain Res..

[24] Karlsson O, Roman E, Brittebo EB (2009). Long-term cognitive impairments in adult rats treated neonatally with beta-N-methylamino-L-alanine. Toxicol. Sci..

[25] Karlsson O (2012). Neonatal exposure to the cyanobacterial toxin BMAA induces changes in protein expression and neurodegeneration in adult hippocampus. Toxicol. Sci..

[26] Karlsson O (2015). Intracellular fibril formation, calcification, and enrichment of chaperones, cytoskeletal, and intermediate filament proteins in the adult hippocampus CA1 following neonatal exposure to the nonprotein amino acid BMAA. Arch. Toxicol..

[27] Karlsson O (2013). Neurotoxin-induced neuropeptide perturbations in striatum of neonatal rats. J. Proteome Res..

[28] Qu Q, Shi Y (2009). Neural stem cells in the developing and adult brains. J. Cell Physiol..

[29] Bose R (2010). Glucocorticoids induce long-lasting effects in neural stem cells resulting in senescence-related alterations. Cell Death Dis..

[30] Tofighi R, Moors M, Bose R, Ibrahim WN, Ceccatelli S (2011). Neural stem cells for developmental neurotoxicity studies. Methods Mol. Biol..

[31] Pierozan P, Jerneren F, Ransome Y, Karlsson O (2017). The choice of euthanasia method affects metabolic serum biomarkers. Basic Clin. Pharmacol. Toxicol..

[32] Jerneren F, Soderquist M, Karlsson O (2015). Post-sampling release of free fatty acids—effects of heat stabilization and methods of euthanasia. J. Pharmacol. Toxicol. Methods.

[33] Pierozan P, Ferreira F, de Lima BO, Pessoa-Pureur R (2015). Quinolinic acid induces disrupts cytoskeletal homeostasis in striatal neurons. Protective role of astrocyte-neuron interaction. J. Neurosci. Res..

[34] Pierozan P, Jerneren F, Karlsson O (2018). Perfluorooctanoic acid (PFOA) exposure promotes proliferation, migration and invasion potential in human breast epithelial cells. Arch. Toxicol..

[35] Song H, Poo M (2001). The cell biology of neuronal navigation. Nat. Cell Biol..

[36] Tilson HA (2000). The role of developmental neurotoxicology studies in risk assessment. Toxicol. Pathol..

[37] Lage S, Annadotter H, Rasmussen U, Rydberg S (2015). Biotransfer of beta-N-methylamino-L-alanine (BMAA) in a eutrophicated freshwater lake. Mar. Drugs.

[38] Main BJ (2018). Detection of the suspected neurotoxin beta-methylamino-l-alanine (BMAA) in cyanobacterial blooms from multiple water bodies in Eastern Australia. Harmful Algae.

[39] Li A (2010). Detection of the neurotoxin BMAA within cyanobacteria isolated from freshwater in China. Toxicon.

[40] Laugeray A (2018). Perinatal exposure to the cyanotoxin beta-N-methylamino-L-alanine (BMAA) results in long-lasting behavioral changes in offspring-potential involvement of DNA damage and oxidative stress. Neurotox. Res..

[41] Karlsson O, Bergquist J, Andersson M (2014). Quality measures of imaging mass spectrometry aids in revealing long-term striatal protein changes induced by neonatal exposure to the cyanobacterial toxin beta-N-methylamino-L-alanine (BMAA). Mol. Cell. Proteom..

[42] Scott, L. L. & Downing, T. G. A single neonatal exposure to BMAA in a rat model produces neuropathology consistent with neurodegenerative diseases. *Toxins.* **10**, 10.3390/toxins10010022 (2017). 10.3390/toxins10010022 PMC5793109 29286334

[43] Pietenpol JA, Stewart ZA (2002). Cell cycle checkpoint signaling: cell cycle arrest versus apoptosis. Toxicology.

[44] Orrenius S, Nicotera P, Zhivotovsky B (2011). Cell death mechanisms and their implications in toxicology. Toxicol. Sci..

[45] Chiu AS (2015). Global cellular responses to beta-methyl-amino-L-alanine (BMAA) by olfactory ensheathing glial cells (OEC). Toxicon.

[46] Kritis AA, Stamoula EG, Paniskaki KA, Vavilis TD (2015). Researching glutamate—induced cytotoxicity in different cell lines: a comparative/collective analysis/study. Front. Cell. Neurosci..

[47] Tseng EE (2010). Glutamate excitotoxicity mediates neuronal apoptosis after hypothermic circulatory arrest. Ann. Thorac. Surg..

[48] Rao SD, Banack SA, Cox PA, Weiss JH (2006). BMAA selectively injures motor neurons via AMPA/kainate receptor activation. Exp. Neurol..

[49] Weiss JH, Koh JY, Choi DW (1989). Neurotoxicity of beta-N-methylamino-L-alanine (BMAA) and beta-N-oxalylamino-L-alanine (BOAA) on cultured cortical neurons. Brain Res..

[50] Lobner D, Piana PM, Salous AK, Peoples RW (2007). Beta-N-methylamino-L-alanine enhances neurotoxicity through multiple mechanisms. Neurobiol. Dis..

[51] Linke SP, Clarkin KC, Di Leonardo A, Tsou A, Wahl GM (1996). A reversible, p53-dependent G0/G1 cell cycle arrest induced by ribonucleotide depletion in the absence of detectable DNA damage. Genes Dev..

[52] Zhuang W (2011). The mechanism of the G0/G1 cell cycle phase arrest induced by activation of PXR in human cells. Biomed. Pharmacother..

[53] Mitew S (2010). Focal demyelination in Alzheimer’s disease and transgenic mouse models. Acta Neuropathol..

[54] Munno DW, Syed NI (2003). Synaptogenesis in the CNS: an odyssey from wiring together to firing together. J. Physiol..

[55] Frimat JP (2010). The network formation assay: a spatially standardized neurite outgrowth analytical display for neurotoxicity screening. Lab Chip.

[56] Tear G (1999). Neuronal guidance. A genetic perspective. Trends Genet..

[57] Wilson MT, Keith CH (1998). Glutamate modulation of dendrite outgrowth: alterations in the distribution of dendritic microtubules. J. Neurosci. Res..

[58] Kossel AH, Williams CV, Schweizer M, Kater SB (1997). Afferent innervation influences the development of dendritic branches and spines via both activity-dependent and non-activity-dependent mechanisms. J. Neurosci..

[59] Maletic-Savatic M, Malinow R, Svoboda K (1999). Rapid dendritic morphogenesis in CA1 hippocampal dendrites induced by synaptic activity. Science.

[60] Wilson MT, Kisaalita WS, Keith CH (2000). Glutamate-induced changes in the pattern of hippocampal dendrite outgrowth: a role for calcium-dependent pathways and the microtubule cytoskeleton. J. Neurobiol..

[61] LoTurco JJ, Blanton MG, Kriegstein AR (1991). Initial expression and endogenous activation of NMDA channels in early neocortical development. J. Neurosci..

[62] Monyer H, Burnashev N, Laurie DJ, Sakmann B, Seeburg PH (1994). Developmental and regional expression in the rat brain and functional properties of four NMDA receptors. Neuron.

[63] Di Giorgi-Gerevini V (2005). Endogenous activation of metabotropic glutamate receptors supports the proliferation and survival of neural progenitor cells. Cell Death Differ..

[64] Erichsen JL, Blaabjerg M, Bogetofte H, Serrano AM, Meyer M (2015). Group I metabotropic glutamate receptors: a potential target for regulation of proliferation and differentiation of an immortalized human neural stem cell line. Basic Clin. Pharmacol. Toxicol..

[65] Modgil S, Lahiri DK, Sharma VL, Anand A (2014). Role of early life exposure and environment on neurodegeneration: implications on brain disorders. Transl. Neurodegener..

[66] Rodier PM (1995). Developing brain as a target of toxicity. Environ. Health Perspect..

[67] Artaglione, A. M., Venerosi, A. & Calamandrei, G. Early-life toxic insults and onset of sporadic neurodegenerative diseases—an overview of experimental studies. *Curr. Top. Behav. Neurosci.* **29**, 231–264 (2016). 10.1007/7854_2015_416 26695168

[68] Graff J, Kim D, Dobbin MM, Tsai LH (2011). Epigenetic regulation of gene expression in physiological and pathological brain processes. Physiol. Rev..

[69] Vaiserman AM (2013). Long-term health consequences of early-life exposure to substance abuse: an epigenetic perspective. J. Dev. Orig. Health Dis..

[70] Tian F, Marini AM, Lipsky RH (2010). NMDA receptor activation induces differential epigenetic modification of Bdnf promoters in hippocampal neurons. Amino Acids.

[71] Cortes-Mendoza J, Diaz de Leon-Guerrero S, Pedraza-Alva G, Perez-Martinez L (2013). Shaping synaptic plasticity: the role of activity-mediated epigenetic regulation on gene transcription. Int. J. Dev. Neurosci..

[72] Lilja T, Heldring N, Hermanson O (2013). Like a rolling histone: epigenetic regulation of neural stem cells and brain development by factors controlling histone acetylation and methylation. Biochim. Biophys. Acta.

[73] Gonzalez-Hunt CP, Wadhwa M, Sanders LH (2018). DNA damage by oxidative stress: measurementstrategies for two genomes. Curr. Opin. toxicol..

[74] Cavaliere F, Benito-Munoz M, Panicker M, Matute C (2013). NMDA modulates oligodendrocyte differentiation of subventricular zone cells through PKC activation. Front Cell Neurosci..

[75] Schneider L (2013). DNA damage in mammalian neural stem cells leads to astrocytic differentiation mediated by BMP2 signaling through JAK-STAT. Stem Cell Rep..

