# Supplementary material for: Mitotically heritable effects of BMAA on striatal neural stem cell proliferation and differentiation
Source: Cell Death Dis. 2019 Jun 17;10(7):478. doi: 10.1038/s41419-019-1710-2 (PMC6579766; doi:10.1038/s41419-019-1710-2)
Supplement: Supplementary file 1 — Supplemental Figure 1 [file 41419_2019_1710_MOESM1_ESM.pdf]

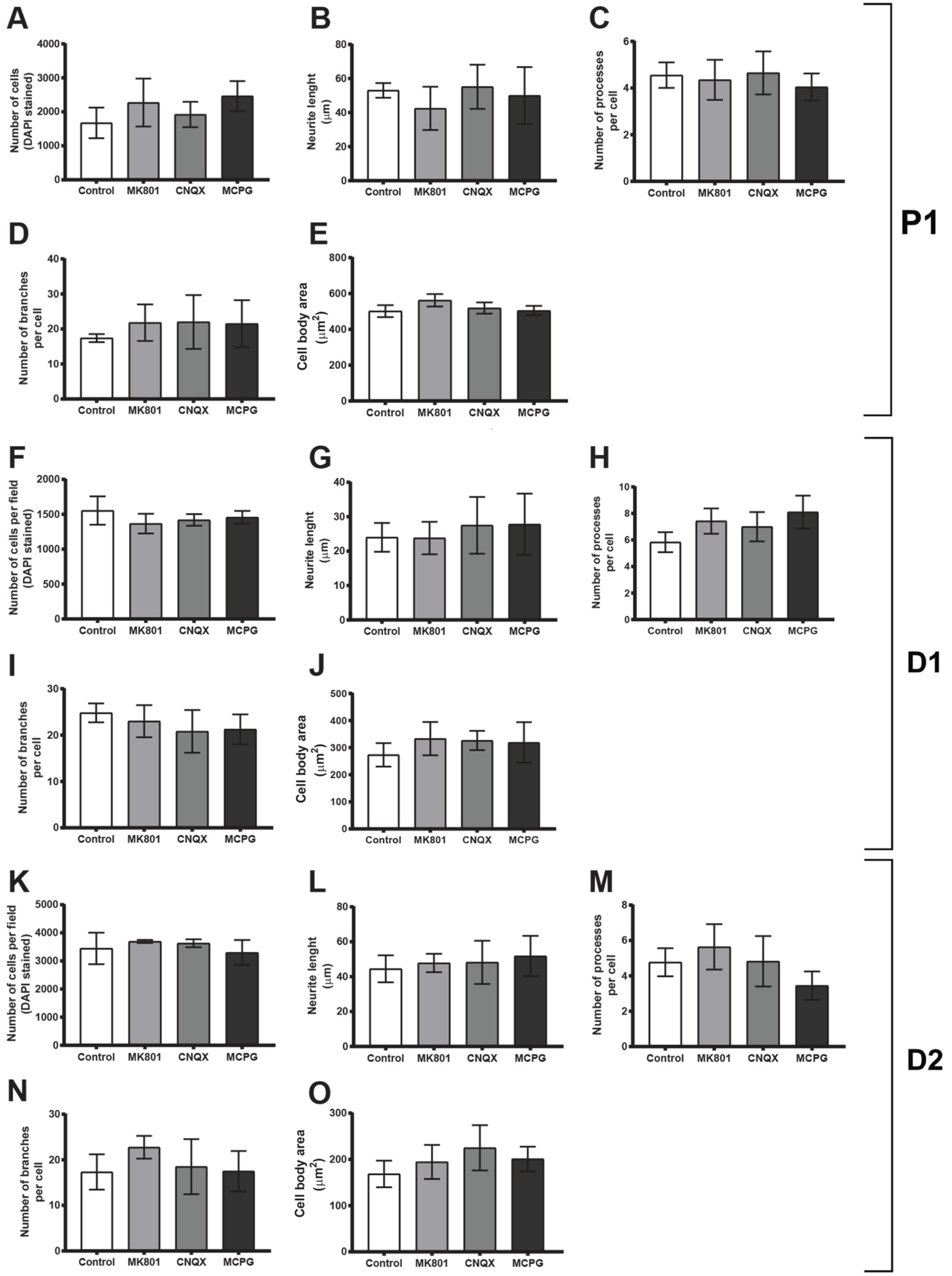

Morphometric analysis of NSCs preincubated with glutamatergic antagonists (100 μM MK-801, 25 μM CNQX or 50 μM MCPG) for 30 min. (A-E) Morphometric analysis of NSCs, (F-J) D1 and (K-O) D2 cells. The images were collected with a 10X objective in an ImageXpress Micro XLS Widefield High-Content analysis System and automatically analyzed with SoftMax Pro Software. Data are reported as mean ± SD for three different experiments and analyzed by two-way ANOVA followed by Tukey-kramer test.
